# The Emerging Roles of Extracellular Vesicles in Osteosarcoma

**DOI:** 10.3389/fonc.2019.01342

**Published:** 2019-12-03

**Authors:** Francesca Perut, Laura Roncuzzi, Nicola Baldini

**Affiliations:** ^1^Laboratory for Orthopedic Pathophysiology and Regenerative Medicine, IRCCS Istituto Ortopedico Rizzoli, Bologna, Italy; ^2^Department of Biomedical and Neuromotor Sciences, University of Bologna, Bologna, Italy

**Keywords:** osteosarcoma, extracellular vesicles, multidrug resistance, liquid biopsy, microenvironment

## Abstract

Extracellular vesicles (EVs) are heterogeneous nanosized vesicles that are constitutively released by virtually all types of cells. They have been isolated in almost all body fluids. EVs cargo consists of various molecules (nucleic acids, proteins, lipids, and metabolites), that can be found on EVs surface and/or in their lumen. EVs structure confer stability and allow the transfer of their cargo to specific cell types over a distance. EVs play a critical role in intercellular communication in physiological and pathological settings. The broadening of knowledge on EVs improved our comprehension of cancer biology as far as tumor development, growth, metastasis, chemoresistance, and treatment are concerned. Increasing evidences suggest that EVs have a significant role in osteosarcoma (OS) development, progression, and metastatic process. The modulation of inflammatory communication pathways by EVs plays a critical role in OS and in other bone-related pathological conditions such as osteoarthritis and rheumatoid arthritis. In this review we describe the emerging data on the role of extracellular vesicles in osteosarcoma and discuss the effects and function of OS-derived EVs focusing on their future applicability in clinical practice.

## Introduction

Extracellular vesicles are lipid bilayer nanovesicles containing nucleic acids (DNA, mRNA, and miRNA), proteins, metabolites and lipids (1). EVs were first described by Johnstone et al., that demonstrated EVs ability to transport transferrin receptor outside the cells during the maturation of reticulocyte (2). Recently, these nanovesicles have gained substantial attention as crucial factors in maintaining normal cellular and biological physiology. These vesicles are proposed to be tailor-made specialized mini-maps of their cell of origin, and have peculiar functions in cell-to-cell communication (3). Extracellular vesicles are a class of nanovesicles including exosomes and microvesicles, that have been defined and sub-grouped on the basis of their size, biogenesis and composition. Exosomes are classically considered as 30–100 nm, they are part of the endosomal compartment and are generated within large intracellular multivesicular bodies. They are released into the extracellular space upon fusion with the plasma membrane. Microvesicles range as 100–1,000 nm and are produced by direct budding from the plasmatic membrane (4).

The EVs lipid membrane protects its cargo from enzymatic degradation making them ideal carriers for local and long-distance transport (3). EVs have been identified in nearly all eukaryotic and prokaryotic cells and are secreted in physiological and pathological conditions (5, 6). They have been isolated from most body fluids including plasma, saliva and urine (7). Furthermore, extracellular vesicles are observed abundantly in tumor microenvironment where they play an important role in signaling pathways (8, 9). The presence of matrix metalloproteinases (MMPs) and MMP regulators in EVs showed their crucial role in extracellular matrix remodeling, that is involved not only in metastatic process but also in several bone-related conditions (10).

## Osteosarcoma

**Figure 1:**
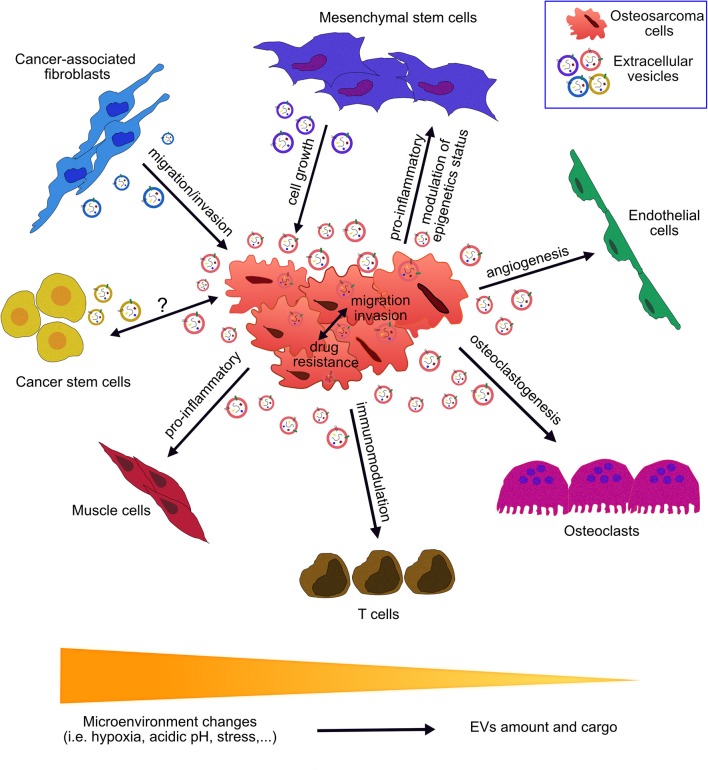
Role of extracellular vesicles in the communication between osteosarcoma cells and the tumor microenvironment. Osteosarcoma cells interact with the surrounding cells through secretion and up-take of extracellular vesicles (EVs). EVs cargo consists of nucleic acids, proteins, lipids, and metabolites. EVs have been found to play roles in a multitude of pathways involved in tumor growth, progression and metastatic process. EV-mediated crosstalk occurs through the trafficking of vesicle-associated components to endothelial cells, osteoclasts, T cells, muscle cells, cancer stem cells, cancer-associated fibroblasts, mesenchymal stem cells, and osteosarcoma cells. OS-derived EVs influence angiogenesis, osteoclastogenesis, immunomodulation, drug resistance, epigenetics status, invasion, and migration processes. EVs derived from cells of OS microenvironment affect OS growth, migration, and invasion. Peculiar microenvironment tumor conditions (acidic pH, hypoxia and stress) affect EVs secretion and features.

Osteosarcoma is the most common primary bone tumor and the more frequent pediatric solid cancer (11). To date, the standard treatment for osteosarcoma is based on neoadjuvant chemotherapy, surgery and post-operative chemotherapy. This aggressive treatment does not guarantee a favorable outcome, principally in patients with metastatic and/or recurrent disease (12, 13), thus new therapies are needed. Therefore, a major translational objective of osteosarcoma research is to identify new therapeutic markers and their clinical significance. In this review, we describe the emerging data on the role of EVs in osteosarcoma growth, metastasis, and chemoresistance (Figure 1), focusing on their future applicability in clinical practice.

## Effects of OS-derived EVs on Cells of Tumor Microenvironment

The specific tumor-driving genetic alterations related to OS development are currently poorly understood. Although there is still no final consensus on the cell of origin for osteosarcoma, the regulation at epigenetic level may be an early event occurring in the transformation of mesenchymal stem cells (MSC) during OS development (14). Recently, Mannerström et al. examined the EV-mediated intercellular crosstalk of MSC and OS. The authors demonstrated that OS-EVs modulate the epigenetic status of MSC, through hypomethylation of long interspersed nuclear element 1. Furthermore, OS-derived EVs influenced the expression of matrix metallopeptidase 1, vascular endothelial growth factor A (VEGF-A), and intercellular adhesion molecule 1 which are related to bone microenvironment remodeling (15).

Tumor growth, progression, and metastatic process are supported by angiogenesis, that guarantees oxygen and nutrient supply to the OS expanding mass, and provides an escape route for tumor cells to enter the circulation and spread to distant organs (16). Different cells and mediators are involved in the angiogenic process. Recently, we highlighted the ability of OS derived-EVs to contribute to tumor angiogenesis. This pro-angiogenic activity is mediated by angiogenesis-related proteins (serpin-E1, serpin-F1, TIMP-1, thrombospondin-1, urokinase-type plasminogen activator (uPA), VEGF, pentraxin-3, PDGF-AA, angiopoietin-2, coagulation factor-III, CD26, CD105, endostatin, endothelin-1, and HB-EGF) and miRNAs (miR-146a-5p, miR-10b-5p, miR-143-3p, miR-382-5p, miR-150-5p, miR-125b-5p, miR-27a-3p, miR-145-5p, miR-26a-5p, miR-93-5p, miR-21-5p, miR-92a-3p, and miR-106a-5p) that have been identified in OS derived-EVs cargo (17). Moreover, Raimondi et al. showed that OS derived-EVs stimulated endothelial cells to express and secrete elevated levels of the pro-angiogenic factor VEGF, and interleukins (IL-6 and IL-8) (18). The role of tumor-derived EVs cargo in stimulation of angiogenesis is well-recognized in other tumors such as multiple myeloma, glioma, renal cell carcinoma, pancreatic, colorectal, prostate, and lung cancer (19). Thus, angiogenic EV cargo can now be reasonably considered a common aggressive trait of cancer derived-EVs.

Osteosarcoma is generally considered a tumor of osteoblastic lineage, and it has been demonstrated that osteoclasts have a crucial role in OS aggressiveness and poor response to chemotherapy (20, 21). Osteoclast formation and bone resorption are stimulated by osteoclast-inducing factors secreted by OS cells themselves, and, additionally, by the pro-osteoclastogenic cargo of OS-derived EVs (18, 22). In particular, the biochemical characterization of OS-derived EVs identified a profile of bioactive pro-osteoclastic factors including matrix metalloproteinase-1 and 13, transforming growth factor β (TGF-β), CD-9 and receptor activator of nuclear factor kappa-β ligand (RANKL) (22). In addition, Raimondi et al. identified a pro-osteoclastic miRNA cargo in OS-derived EVs. The authors demonstrated that EVs contain miR-148a-3p and miR-21-5p, known for their involvement in the tumor microenvironment establishment (18). The functional role of miR-148a-3p in bone homeostasis, osteoclastogenesis and bone metabolism *in vivo* has been previously described (23). Additionally, miR-21-5p has been found highly expressed in osteoclast precursors, and upregulated during RANKL-induced osteoclastogenesis (24). These studies suggest a specific role of the pro-osteoclastogenic cargo of EVs in the alteration of bone remodeling homeostasis in OS bone microenvironment.

The contribution of EVs in tumor progression and metastatic process may be exerted through both local and distant intercellular communication. Macklin et al. demonstrated a role of EVs as mediators in the transfer of migratory and invasive characteristics from OS subclones with highly metastatic traits to poor metastatic cells (25). The hypothesis that a local interclonal cooperation through EV production and transfer favor the metastatic progression of OS, and can determine organotropic metastasis by inducing a pre-metastatic niche, has already been previously demonstrated for human breast and pancreatic cancer (26).

OS derived-EVs may furthermore contribute to metastatic process by prompting MSC to acquire a pro-tumorigenic and pro-metastatic phenotype. Indeed, OS derived-EVs cells selectively incorporate a membrane-associated form of TGF-β, which induces the pro-inflammatory IL-6 production by MSC. MSC-derived IL-6 increases tumor growth and metastasis formation in mice bearing osteosarcoma (8, 27). Moreover, OS cells secreted both the soluble form of uPA and uPA-containing exosomes. Interestingly, the autocrine and paracrine activation of the uPA/uPAR axis has been related to the conversion of OS cells to a metastatic phenotype (28).

The interaction of OS cells with the surrounding immune cells has been explored to support immunotherapy approaches for OS and their potential use as adjuvant therapies (29). The analysis of the proteomic composition of OS-derived EVs in a canine osteosarcoma model identified immunosuppressive proteins with immunomodulatory effects on T cells. In particular, authors demonstrated a diminished activation and proliferation of CD4+ and CD8+ T cells (30). Cancer derived-EVs have been linked with several mechanisms that support tumor development through immune escape (31, 32). In particular, the activation of the programmed death ligand 1 (PD-L1) pathway is used by cancer cells in the process of immune surveillance evasion, and the overexpression of PD-L1 has been associated with increased metastasis in OS (33). Interestingly, Chen et al. showed that metastatic melanoma released EVs carrying PD-L1 that can be used to predict the effect of anti-PD-1 therapy (32).

OS-derived EVs may also play a role in sarcoma-associated cachexia, secondary to aggressive surgical treatment of OS. Interestingly, Mu et al. have related inflammation and the crosstalk between NF-kB and Notch signaling to skeletal muscle atrophy seen in cancer cachexia, and demonstrated that EVs derived from OS murine cells may transfer Notch-activating signals to muscle cells (34).

So far, there are no data available on EVs derived from OS cancer stem cells (CSC), that represent a sub-type of tumor cells with well-known roles in tumor propagation, therapy resistance, recurrence, and metastasis (35). In prostate cancer a different miRNA pattern was found in EV cargo derived from tumor bulk and CSC, thus unveiling additional potential biomarkers and therapeutic targets (36). In this direction, we may assume that future investigations of EV cargo derived from OS CSC will enrich the panel of EV-derived biomarkers.

To summarize, different specific activities of OS-derived EVs on tumor microenvironment cells have been demonstrated. However, according to Jerez et al. the enrichment of EV proteins and miRNA derived from different OS cell lines is heterogeneous (37, 38). This has to be carefully considered when general conclusion on OS behavior are based on a specific miRNAs or protein identified in EV cargo derived by a specific cell line. In order to substantiate the findings, use of additional cell lines and, especially possibly, primary OS cells should be considered in future experiments.

## Functions of EVs Derived From Cells of OS Microenvironment

MSC and cancer-associated fibroblasts (CAFs) are known to support tumor progression and chemoresistance, through paracrine cross-feeding and vesicles secretion (39). Thus, a growing interest has been devoted to explore the activity of EVs derived from MSC or CAF on tumor cells. The transfer of EVs derived miRNA represents an additional level of intercellular communication between stroma and cancer cells. Recently, Wang et al. demonstrated that miR-1228 encapsulated in CAF-derived EVs promotes OS cells migration and invasion by downregulating suppressor cancer cell invasion mRNA expression (40). Recent findings suggest that the contribution of MSC-derived EVs to OS growth is mediated by the activation of Hedgehog signaling pathway (41). Moreover, Lin et al. demonstrated that MSC-derived microvesicles support U2OS cell growth under hypoxia, and that this activity was partially related with the PI3K/AKT and HIF-1αpathways (42).

However, MSC and CAF are not the unique cellular component of osteosarcoma microenvironment. Indeed, osteoblasts, osteoclasts, endothelial, and immune cells coexist with cancer cells and participate in their growth and survival. Moreover, their ability to secrete EVs has already been demonstrated in other pathophysiological models (43–45). Further investigation into how EVs derived from microenvironment cells may act on OS cells will help to elucidate new factors and mechanisms involved in cell communication within tumor microenvironment, and to discover new targets and/or biomarkers.

## Influence of Microenvironment on EVs Secretion and Function

Several microenvironment parameters (acidosis, hypoxia, and elevated interstitial fluid pressure) influence tumor cell viability, proliferation, motility and metabolism, and are able to lead to more aggressive behavior of OS cells (46). Malignant lesions of mesenchymal tumors appear to be quite acidic (47), and it has been demonstrated that extracellular acidosis contributes to OS behavior, chemoresistance, and response to therapy (48–50). Interestingly, Logozzi et al. demonstrated that an acid extracellular pH (6.5) induced a significant increase in EV release, while buffering the medium reduced the EV release in prostate, melanoma, osteosarcoma, breast, adenocarcinoma, and colorectal carcinoma cells (51). According to this, we recently demonstrated an increased amount of EV protein mass secreted by OS cells grown in acidic compared to neutral pH, and a higher pro-angiogenic activity of acidic OS-derived EVs on *in vivo* assay (17). Different hypothesis have been discussed to determine how EVs cargo is transferred from cell to cell, without reaching a conclusive and accepted theory (52). Despite this, Parolini et al. demonstrated an increased fusion efficiency of EVs secreted at low extracellular pH by melanoma cells, possibly due to the modified lipid composition of EVs detected at acidic pH (53). The pharmacological handling of the extracellular and intracellular pH of cancers, that has been considered as a potential additional treatment in tumor therapy (54), may thus also interfere with EVs release and fusion efficiency.

Stress conditions are likewise able to modify the behavior of cells of tumor microenvironment. It is well-known that hypoxia, poor nutrient conditions, and mechanical stress influence MSC secretome (55–57). In this context, it is not surprising that EVs isolated from serum deprived MSC carries tumor supportive miRNA and lncRNA, and increase OS survival and resistance to apoptosis (58, 59).

As microenvironment parameters influence EVs release and cargo, an emerging concept is to produce more sophisticated *in vitro* models to better resemble *in vivo* cell environment when studying EVs, as it is already a well-recognized approach in drug discovery (60). According to this, Villasante et al. pointed out that 3-dimensionality and stiffness of a tumor matrix can determine the size and cargo of EVs released by Ewing's sarcoma cells. These authors suggested to study EVs in 3D rather than in 2D setting to better mimic the native structure of the tumor (61).

## Role of OS-derived EVs in Anti-Cancer Drug Resistance

Multidrug resistance (MDR), intrinsic or acquired, remains a major obstacle to successful osteosarcoma treatment and contributes to poor clinical outcome (62). Recent studies support EVs as playing a key role in OS drug-resistance (63–66). We demonstrated that the MDR phenotype can be induced in OS cells through MDR OS-derived EVs. These nanovesicles are able to decrease OS cell sensitivity to doxorubicin by the transfer of functional MDR-1 mRNA, and its product P-glycoprotein, inducing MDR phenotype to OS doxorubicin-sensitive cells (65). Recently, pre-clinical and clinical data linked EVs to MDR also in hematological malignancies, glioblastoma, neuroblastoma, melanoma, breast, prostate, lung, ovarian, colorectal, gastric, pancreatic, and kidney cancer (66, 67).

Moreover, EVs can mediate MDR through the transfer of specific bioactive molecules including, prosurvival/apoptosis related-factors, and non-coding RNAs (68). Much attention has focused on the miRNAs identified in EVs cargo due to their capacity to interfere in gene regulation and subsequently to be involved in a variety of drug resistance pathways and mechanisms (69). Therefore, the EV and its molecular cargo can be viewed as a fundamental mediator of cancer drug resistance.

## EVs as Biomarkers in OS

Liquid biopsy strategies are now being explored to discover and validate new and more efficient and/or complementary approaches to improve OS diagnosis, management and treatment (70). Liquid biopsies can be profitably used to assess molecular heterogeneity of OS tumors, and to provide dynamic tumor information. In this context, EVs represent a promising target as they can be easily non-invasively isolated from accessible body fluids including blood, urine and saliva. Furthermore, EV cargo is protected from degradation inside a membranous structure, that provides stability and allows prolonged periods of storage of EVs before analysis, making their clinical use feasible (71). EVs contains nucleic acids, proteins, lipids and metabolites that can be identified, characterized and thus used as biomarkers. As far as EV-associated proteins are concerned, circulating levels of EV-associated TGF-β have been found to be increased in osteosarcoma patients, when compared to healthy control subjects (27). A proteomic investigation of circulating EVs in canine serum samples identified EV related proteins useful to distinguish serum of osteosarcoma from serum of healthy or fractured dogs (72). Furthermore, Brady et al. identified two proteins associated with EVs (serpinD1 and MHC class III-complement C6) which allow to discriminate serum derived from different disease stages of OS (72).

The presence of a specific collection of RNAs in EVs cargo may also serve as new or supplementary biomarker in OS diagnosis and progression. Xu et al. showed dysregulated levels of several miRNAs and mRNAs in EVs isolated from serum of OS patients with a poor chemotherapeutic response when compared with good responders (73). Moreover, Bao et al. demonstrated in a pilot study, an increased tumor mutation burden in RNA isolated from metastatic EVs plasma samples compared to non-metastatic ones (74). A prospective observational study to reveal the roles of circulating EVs RNA in lung metastases of primary high–grade osteosarcoma was launched in 2017 and recruiting is still ongoing (ClinicalTrials.gov: Identifier: NCT03108677).

In this review different EVs cargo components have been described as potential biomarkers in OS patients. In Table 1 biomarkers identified in circulating EVs in osteosarcoma are reported. The number of patients and source of EVs (plasma, serum, or blood) were described. These studies were carried out in small cohorts of patients utilizing different methods to isolate EVs. According to Ayers et al. several parameters and challenges will have to be considered before a diagnostic clinical application of EVs can become a solid reality (75). The major concern of all studies using EVs are focused on standardization and improvement of methods to isolate EVs and, if appropriate, to distinguish EV subpopulations. It has to be pointed out that also a standardization of pre-analytical variables is also required to ensure that the quantity and characteristics of EVs reported can be reliably evaluated. The possibility to merge data coming from different laboratories would be simplified by following shared protocols and guidelines. In this respect the International Society of Extracellular Vesicles supported several initiatives to favor method homogenization, such as the EV Transparent Reporting and Centralizing Knowledge (76), the Minimal Information for Studies of EVs (4) and the Clinical Wrap-Up session at ISEV2018 (77). As far as EVs isolation methods are concerned, microfluidic miniaturized systems have recently emerged as promising technology to address both isolation and analysis of EVs in clinical settings, where a small amount of samples are available and rare molecular targets have to be detected (78, 79).

**Table 1:** Biomarkers identified in circulating EVs in osteosarcoma.

**Biomarker**	**Sample Type**	**Number of patients analyzed**	**References**
EV-associated TGFβ	Human serum	*n* = 10 OS patients *n* = 10 healthy donors	(24)
EV-associated SERPING1, HEL-S-71p, HBB, KRT10, HEL180, TIH1, IGLC7, DC33, and characterized protein	Canine serum	*n* = 8 OS group *n* = 5 healthy dogs with traumatic bone fractures *n* = 5 healthy, size-matched controls	(68)
EV-associated SERPIND1 and class III MHC	Canine serum	*n* = 5 OS diagnosis *n* = 5, 2 weeks after amputation *n* = 5 onset of lung metastases	(68)
Serum exosomal miRNAs: miR-124, miR133a, miR-9, miR199a-3p, miR-385, miR-135b, miR-148a, miR-27a Serum exosomal mRNAs: Annexin2, Smad2, MTAP, CIP4, PEDF, WWOX, Cdc5L, P27	Human serum	*n* = 48 OS patients with poor chemotherapeutic *n* = 45 OS patients with good chemotherapeutic response *n* = 51 healthy donors	(69)
EVRNA carries aberrant gene fusions	Human plasma	*n* = 3 OS presurgery patients *n* = 3 OS metastatic patients	(70)
Mutations of RNA in circulating EVs	Human blood	*n* = 40 metastatic and non-metastatic patients Recruiting is still ongoing.	ClinicalTrials.gov Identifier: NCT03108677

## Conclusions

Osteosarcoma is a complex system in which cancer cells, cancer stem cells, mesenchymal cells, immune cells, fibroblasts and endothelial cells coexist and communicate. Recently acquired knowledge indicates that the interactions among these cells are also mediated by extracellular vesicles. The transfer of tumor-supportive traits from osteosarcoma cells to donor cells contribute to cancer growth and metastatic process. On the other hand, EVs derived from microenvironment cells are able to influence tumor growth and migration. Additionally, specific tumor microenvironment features (stress, hypoxia, acidic pH) interfere with EV secretion and cargo. Overall, data from literature suggest multiple functions of EVs in osteosarcoma, paving the way to discover new therapeutic targets and to design innovative diagnostic assays. Future efforts must focus on technological advances in EV purification and characterization to improve EV detection and cargo evaluation in clinical setting. Moreover, one additional challenge for the future will be to associate new functions to subclasses of EVs to identify peculiar pathways involving EVs, and changes in their cargo related to OS stage or response to therapy.

## Author Contributions

All authors listed have made a substantial, direct and intellectual contribution to the work, and approved it for publication.

### Conflict of Interest

The authors declare that the research was conducted in the absence of any commercial or financial relationships that could be construed as a potential conflict of interest.
